# MediaDB: A Database of Microbial Growth Conditions in Defined Media

**DOI:** 10.1371/journal.pone.0103548

**Published:** 2014-08-06

**Authors:** Matthew A. Richards, Victor Cassen, Benjamin D. Heavner, Nassim E. Ajami, Andrea Herrmann, Evangelos Simeonidis, Nathan D. Price

**Affiliations:** 1 Department of Chemical and Biomolecular Engineering, University of Illinois at Urbana-Champaign, Urbana, Illinois, United States of America; 2 Institute for Systems Biology, Seattle, Washington, United States of America; 3 Luxembourg Centre for Systems Biomedicine, University of Luxembourg, Esch-sur-Alzette, Luxembourg; Virginia Commonwealth University, United States of America

## Abstract

Isolating pure microbial cultures and cultivating them in the laboratory on defined media is used to more fully characterize the metabolism and physiology of organisms. However, identifying an appropriate growth medium for a novel isolate remains a challenging task. Even organisms with sequenced and annotated genomes can be difficult to grow, despite our ability to build genome-scale metabolic networks that connect genomic data with metabolic function. The scientific literature is scattered with information about defined growth media used successfully for cultivating a wide variety of organisms, but to date there exists no centralized repository to inform efforts to cultivate less characterized organisms by bridging the gap between genomic data and compound composition for growth media. Here we present MediaDB, a manually curated database of defined media that have been used for cultivating organisms with sequenced genomes, with an emphasis on organisms with metabolic network models. The database is accessible online, can be queried by keyword searches or downloaded in its entirety, and can generate exportable individual media formulation files. The data assembled in MediaDB facilitate comparative studies of organism growth media, serve as a starting point for formulating novel growth media, and contribute to formulating media for *in silico* investigation of metabolic networks. MediaDB is freely available for public use at https://mediadb.systemsbiology.net.

## Introduction

Genomic and high-throughput sequencing technologies enable the generation of large amounts of genetic information on microorganisms without the need to grow cultures in the lab. Armed with these technologies, we can automatically generate draft metabolic network reconstructions for organisms directly from genome annotations [1] and derive metabolic network models to simulate microbial growth *in silico*. These models can be improved through an iterative curation process between experimental and computational investigations [2]. To date, this iterative process has been most successfully advanced by partnering *in silico* reconstruction with *in vitro* characterization of isolates grown in defined laboratory media—an experimental approach that remains the most comprehensive method for characterizing microbial physiology [3]–[9]. Techniques for building metabolic network reconstructions from genomic data have progressed sufficiently to enable the application of *in silico* models for characterizing microbes that have not been cultivated *in vitro*.

Only 0.1–1% of the estimated number of microbial species have been isolated and successfully cultivated in a laboratory environment [5], [6], [8]. The collection of species we can currently culture spans only 30 of over 100 established phyla and mostly contains fast-growing organisms—organisms that are not the most prevalent species in the environment [7], [9]. A range of novel techniques have been applied in efforts to culture less characterized microbes, such as using diffusion chambers to mimic environmental conditions [10]–[13], adding growth factors or signaling compounds secreted from other organisms [14]–[17], diluting media nutrients to lower concentrations [18]–[24], increasing incubation time [19], [20], [23], [25]–[28] and running high-throughput cultures [21], [29]–[31]. These innovations have increased the diversity and number of culturable organisms, but the large number of factors that can affect *in vitro* growth still presents a challenge for isolating and culturing microbes from environmental samples.

Recently, computational modeling has been successfully applied to support culturing efforts. Several groups have used metabolic reconstructions, which are based on organism-specific genome sequence and biochemical knowledge, to assist in media design. Applications of these networks to media design have included both direct querying of the metabolic network to identify key metabolites for growth media design [32] and simulating growth on different substrates with a genome-scale metabolic model to predict media formulation [33]. Efforts that use a metabolic network model must define an *in silico* medium to enable calculations such as Flux Balance Analysis (FBA) [34]–[36]. The model and simulated medium then are iteratively refined until the network successfully predicts biomass production.

Thus, simulating growth of an uncultured organism with a metabolic model requires the definition of an *in silico* growth medium or a set of candidate media, which may then be validated *in vitro*. The definition of a growth medium *in silico* often begins in the same fashion as *in vitro* attempts: by starting with a medium that has supported simulated growth in models of organisms related to the desired isolate. However, this approach is complicated by the fragmentation of information in the literature. To overcome this obstacle, we have created MediaDB: a database of experimentally determined, chemically defined growth media conditions that aims to support efforts to leverage -omics data and modeling techniques for characterizing previously uncultured isolates. MediaDB is a manually curated database of defined media formulations for organisms with fully sequenced genomes, emphasizes organisms that have existing metabolic network models, and is the first publically available electronic resource that specifically brings together organisms with genomic data and their associated growth media. MediaDB will facilitate investigation of the relationship between microbial genomes and media composition, serving as both a central repository of data linking genome sequence to media compositions, and as a resource that facilitates model-supported design of cultivation media.

## Database Construction and Content

**Figure 1:**
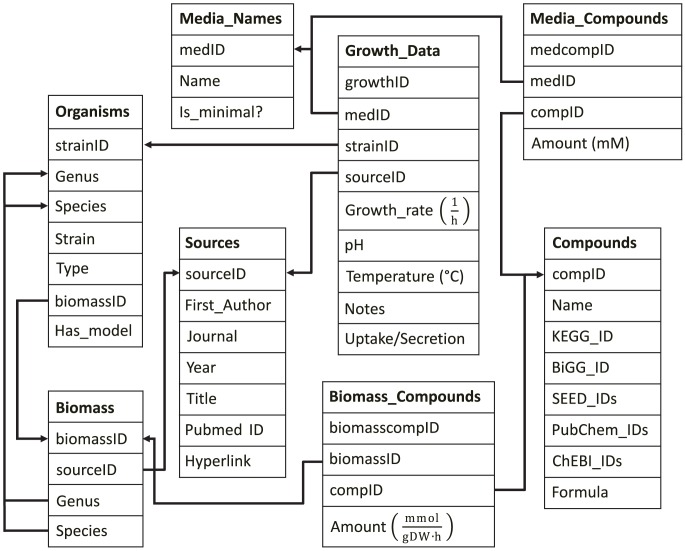
Simplified database schema. This graph shows the connections between the 6 main tables, Organisms, Compounds, Media_Names, Biomass, Sources, and Growth_Data. Also shown are Media_Compounds and Biomass_Compounds, linking tables that connect the Compounds table to the Media_Names and Biomass tables, respectively. Arrows indicate foreign key relationships, in which the head of the arrow points to the primary key being referenced. A full map of the MediaDB schema containing all tables and their connections can be found in Figure S1.

All data in MediaDB were manually curated from existing primary literature sources. We conducted organism-by-organism literature searches using standard search engines—Google Scholar, PubMed, Web of Science—on the list of *in silico* organisms maintained by the Systems Biology Research Group at UCSD [37]. Our searches were aimed at finding experimentally-verified growth data on defined media for as many organisms with curated metabolic models as possible. The search results were curated manually and the media related information was extracted and formatted in the MediaDB schema, a MySQL database consisting of 12 tables and constructed around 6 main data tables: Organisms, Compounds, Media_Names, Biomass, Sources, and Growth_Data (Figure 1). The full schema is included as supporting information (Figure S1).

### Organisms

The Organisms table includes fields for genus, species, and strain, a “type” designation that specifies the organism's kingdom classification, a Boolean value denoting whether the organism has been modeled *in silico*, and, if applicable, a link to the biomass composition for that organism. As shown in Table 1, MediaDB currently contains 208 unique Organisms objects spanning 57 species and 46 genera.

**Table 1:** Taxonomy of organisms currently in MediaDB.

Kingdom	Genera	Species	Strains
*Archaea*	*4*	*5*	*14*
*Bacteria*	*36*	*43*	*172*
*Eukaryote*	*6*	*9*	*22*
**TOTAL**	**46**	**57**	**208**

Bacteria make up the majority of organisms in the database, reflecting the distribution of species that have been cultured in the laboratory and the MediaDB's emphasis on organisms with existing *in silico* metabolic reconstructions. Such reconstructions exist for 39 of the 43 bacterial species and 51 of the 57 total species in the database. The database also includes many strains for model organisms; *Escherichia coli* and *Bacillus subtilis* contribute 54 and 16 bacterial strains, respectively, to the database.

### Compounds

The Compounds table includes fields to describe a chemically-defined compound in terms of its common names, chemical formula, and identifiers that can be used to cross-reference with other databases (KEGG, BiGG, Seed, ChEBI and PubChem) [38]–[43]. We included identifiers from these databases to enable easier exchange of information between researchers, enhance compatibility with commonly-used resources, and ease development of automated computational analyses that use data in MediaDB. Of the 14,795 compounds contained in the database, 14,785 (99.9%) have identifiers from at least one other database.

Unlike the other tables in the MediaDB schema, the Compounds table was initially curated based on the KEGG database rather than from specific literature sources and was supplemented with manual entries from other databases as necessary. Its primary purpose is to describe the composition of other data types (Media_Names, Biomass).

### Media_Names

The Media_Names table consists of fields specifying a media formulation with a descriptive name, a Boolean value indicating whether or not the particular media formulation was described as minimal in its source material, and a list of names and amounts of each compound that makes up that medium in units of millimolar (mM). Due to the many-to-many nature of relating compounds to different media compositions, the relationship between media formulations and compounds are contained within the Media_Compounds table, but can be queried to find the compounds that make up a particular media formulation. MediaDB only contains chemically defined media formulations and does not include complex formulations, such as media that use yeast extract. The focus on chemically defined media was selected to facilitate computational simulation of growth conditions and to support efforts to cultivate uncultured organisms in the laboratory. MediaDB currently contains 461 different media formulations.

### Biomass

The Biomass table consists of fields describing the compounds included in the biomass objective function used in FBA of metabolic network models to simulate exponential cell growth and contains organism genus and species, the list of compounds present in the biomass composition, and the stoichiometric coefficient of each compound in relation to one “unit” of biomass. Like the MediaDB description of media, biomass is also specified by the compounds that make up its composition, resulting in a many-to-many relationship. The Biomass_Compounds table contains the links between biomass compositions and compounds and can be queried to find the compounds that make up a particular biomass composition.

As detailed in Thiele *et al.* [2], the biomass composition is an important objective function for FBA of metabolic network models; however, it can also be difficult to experimentally determine detailed biomass composition for an organism. Thus, the biomass composition is a salient factor to consider in model construction and refinement, but we found few unique examples of this data type in existing literature sources. Instead, many models have defined the organism biomass composition by using or slightly modifying the biomass objective function from another model. We have included 4 different biomass compositions in MediaDB to provide a basis for users to construct biomass compositions for their own organisms by refining established ones.

### Sources

The Sources table consists of fields describing a primary literature source (usually a book or a journal article) and is specified using the first author's last name, the title of the work, the journal, the year of publication and, if applicable, the PubMed identifier and URL to the article. Sources are added to MediaDB if they report experimental laboratory growth of an organism in MediaDB in a medium in MediaDB. MediaDB currently contains 147 unique sources that directly link to any experimental growth media information they provided.

### Growth Data

The Growth_Data table describes the combination of physical parameters reported by a literature source for *in vitro* growth of a specific organism. The Growth_Data table links the tables describing an organism, medium, and literature source, and adds information about temperature, pH, growth rate, product secretion rates, and nutrient uptake rates (whenever reported in the literature source). MediaDB currently contains 765 growth conditions.

In many instances, we found rate data associated with a particular growth condition in the form of an experimentally-measured growth rate (*μ*) measured in *h* ^−1^. We stored growth rates in the Growth_Data data field, thereby providing quantitative measures to assist in future metabolic model development. Some growth conditions were also reported with other growth-associated measurements: product secretion rates, medium compound uptake rates and product yields. Unlike growth rates, a growth condition could be associated with multiple measurements of secretion/uptake/yield; hence, we created the Secretion_Uptake table to house these rates and link them to their growth conditions. MediaDB currently contains 557 measured growth rates, 49 metabolite uptake rates, 22 product secretion rates, and 58 product yield coefficients.

## Website Construction and Navigation

The MediaDB website (https://mediadb.systemsbiology.net/) provides a user-friendly interface for performing the two main functions of our database: data browsing and exporting.

### Data browsing

**Figure 2:**
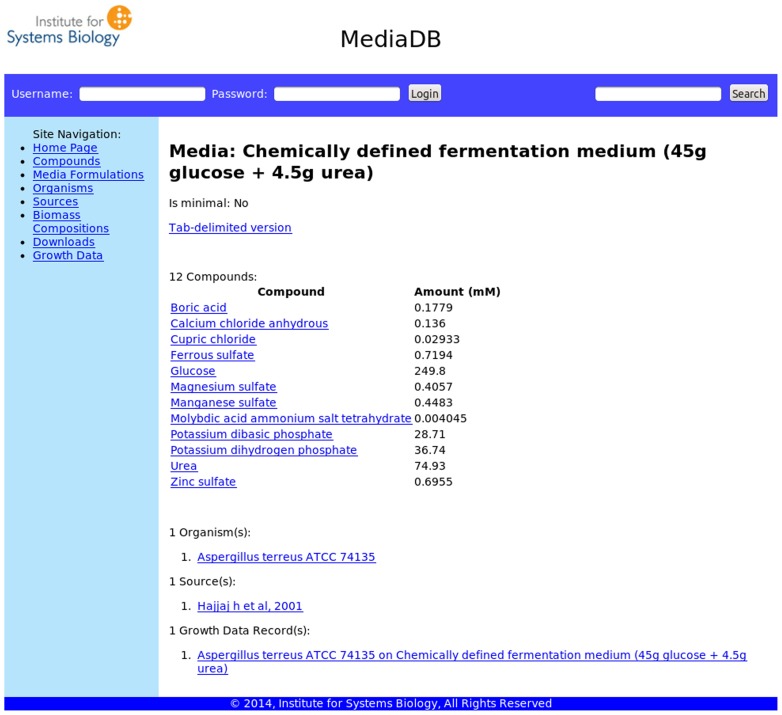
The MediaDB website. The database can be found at https://mediadb.systemsbiology.net. This page shows the composition of a media formulation and displays links to the organism, source, and growth record that use this medium. The “Site Navigation” panel lists the different tables that can be browsed manually and also the “Downloads” tab, where the user can export a copy of the entire MediaDB schema. The search field is at the top right of the page.

Browsing allows the user to query MediaDB with provided data type categories, to manually search through information by navigating through the different data tables or to use keywords to search through the parameters that specify the growth condition entries (see Figure 2). The search function matches the given keyword to data entries in all tables and returns the results sorted by the table that contains the matched record.

Tables in the database are linked together on the webpage by cross-referencing to better display all pertinent information for each entry. For example, an entry in the Organisms table shows all of the related growth condition entries collected for that organism, including links to the literature source entries. Similarly, each media formulation entry links to entries for all the compounds present in that media formulation, all of the organisms reported to grow in that media formulation, and the literature source entries where the media formulation was reported. A Compounds entry displays links to all the media formulations in which the compound appears. A Source entry displays links to all the growth conditions reported in that source, as well as links to the online version of that source, when applicable.

### Data export

Data can be exported from MediaDB in two different ways, allowing the user flexibility in deciding what information is important for their particular project. The most basic export, found under “Downloads” on the webpage, allows the user to download a copy of the entire MediaDB schema and all database entries to use independently of the website. This option allows the most flexibility in dealing with the data, but requires that the user be familiar enough with relational database management in MySQL to use the SQL file generated by this export.

The second export option is individual media formulation or biomass composition download, available on each media formulation or biomass composition entry page under “Tab-delimited version”. This option generates a tab-delimited text file with a list of compounds and their concentrations in the chosen media formulation or biomass composition. The file also includes identifiers for the compounds in other databases. These identifiers facilitate cross-referencing of the various metabolite identifiers used in different *in silico* metabolic network models.

## Database Utility

### Statistics for compounds

Because the MediaDB schema provides links between organisms and the compounds in their growth media, it enables investigation of media components across organisms. For example, we compiled a list of every chemical compound that appears at least once in a growth medium for all 57 species in the database (see Table S1 for full results). Out of 260 unique compounds, the most commonly occurring compound across all species was calcium chloride (CaCl_2_), a salt that appears in the growth media of 49 species (86% of all species in MediaDB), because it is often included in stock trace element/mineral solutions. Salts accounted for nine of the top ten most frequent compounds with the only exception being biotin, a vitamin that often appears in stock vitamin solutions and was present for 29 species (51%). Other components of media, such as the carbon source and amino acids, were less uniform across species; the most common carbon source and amino acid were glucose (47%) and cysteine (37%), respectively (a list of the most frequent compounds is shown in Table 2).

**Table 2:** Highest frequency compounds in MediaDB.

Top 10 Compounds		Top 5 Carbon Sources	
CaCl2	86%	Glucose	47%
MgSO4	72%	Acetate	33%
KH2PO4	65%	Glycerol	19%
NaCl	65%	Pyruvate	12%
FeSO4	61%	Ethanol/Succinate	9%
ZnSO4	61%	**Top 5 Amino Acids**	
K2HPO4	58%	Cysteine	37%
NH4Cl	53%	Aspartate	33%
Biotin	51%	Arginine	33%
CuSO4	49%	Glutamate	32%
		Leucine	32%

Percentages reflect the fraction of species that contain each compound in at least one growth medium.

Our analysis also identified the *least* common compounds in media; 97 of the 260 compounds (37%) appeared in media for only one species and 139 (53%) appeared in media for one or two species only. These uncommon compounds generally fell into one of the following categories: 1) Trace metals included in stock solutions (e.g., nickel sulfate for *Shewanella oneidensis*); 2) Buffers for pH maintenance (e.g., ACES for *Mycobacterium tuberculosis*); 3) Antibiotics used to select for mutant strains (e.g., kanamycin for *Synechocystis PCC6803*); 4) Uncommon carbon sources (e.g., galactose for *Streptomyces coelicolor*); 5) Alternate vitamin forms (e.g., sodium pantothenate rather than calcium pantothenate for *Haemophilus influenzae*); 6) Compounds that fit niche organism metabolisms (e.g., 2-mercaptoethanesulfonate for *Methanococcus maripaludis*). Compounds in the final category were of particular interest, because they could be tied to unique portions of the known metabolism of the organism. For example, 2-mercaptoethanesulfonate (coenzyme M) only appears in media for the methanogen *M. maripaludis*, because it is a vital cofactor involved in methane production for that organism. As MediaDB grows, we expect that identifying such unusual compounds will play an increasingly useful role in media design.

### Linking growth media to metabolism

MediaDB provides a framework for comparing the nutritional requirements of different organisms and currently includes information on a range of microbes, with a focus on organisms that have been modeled *in silico*. In order to demonstrate how MediaDB supports such comparative analysis, we compared media formulations for two organisms that have metabolic network models: *E. coli*, a model bacterium that has been grown with a wide range of compounds (81 different compounds), and *Methanosarcina acetivorans*, a model archaeon that has been grown using a smaller range of compounds (12 different compounds).

Seven compounds appeared in media formulations for both organisms: one carbon source (acetate) and six simple salts (NH_4_Cl, CaCl_2_, MgCl_2_, KCl, KH_2_PO_4_, NaCl). The compounds unique to *E. coli* included multiple 5- or 6-carbon sugars (e.g., glucose, lactose, fructose, and succinate) and 19 of the 20 standard L-form amino acids (all except cysteine). The 5 compounds unique to *M. acetivorans* included methanol, a simple carbon source for methanogens that rarely appears in media for other organisms (fellow methanogen *Methanosarcina barkeri* and pathogen *Candida glabrata* are the only other species in MediaDB with media that include methanol). We also observed that, in contrast to the *E. coli* media data, cysteine was the only amino acid that appeared in growth media for *M. acetivorans*.

We expanded our comparison by using manually curated metabolic models for both *E. coli* [44] and *M. acetivorans* [45] to examine the differences found in media compounds. By examining reactions in the models, we observed that the model for *E. coli* included uptake pathways for many carbon sources that are absent in the *M. acetivorans* model, including all of the carbon sources reported in MediaDB. The *E. coli* model predicted that methanol could be produced during growth, but not consumed, whereas the *M. acetivorans* model predicted the ability to consume methanol for growth and methane production. The models also provided mechanistic justification for our media analysis that suggested differences in cysteine metabolism; the *M. acetivorans* model had the ability to both consume and secrete cysteine and the *E. coli* model predicted cysteine secretion, but not consumption. We extended this analysis by testing the models for growth on a range of experimental media from the database. We selected 11 media for *E. coli*—one for each carbon source—and the one medium for *M. acetivorans* in MediaDB, then simulated each model for growth on all 12 media (see File S1 for an example of this procedure). The *E. coli* model predicted growth on all 12 media, mirroring the organism's versatility to grow on many different carbon sources. The *M. acetivorans* model required modification to remove trace metals from the biomass objective function in order to predict growth on any medium. After the trace metals (which are not included in simulated *E. coli* media) were removed from the *M. acetivorans* model objective function, it accurately predicted growth on its own medium and on the *E. coli* medium with acetate as the carbon source, but not on any of the other media, reflecting the organism's inability to grow on complex carbon sources.

This case study illustrates the use of MediaDB as a tool for investigating the differences in nutritional requirements between organisms and as a source for *in silico* medium formulation. The differences between cultivation media for *E. coli* and *M. acetivorans* were identified using MediaDB and explained using the organisms' respective metabolic models, which include fundamental differences in carbon source and amino acid metabolism. In this example, the results of the comparisons between the media sources and metabolic models were quite parallel, as expected, because both models were manually constructed based on genomic information and information from the primary literature, including media formulation sources. In other cases, where there is disagreement between model simulation results and media information reported, MediaDB will support efforts to improve metabolic network reconstruction by providing information regarding experimentally determined media conditions.

### Organism clustering by compound similarity

**Figure 3:**
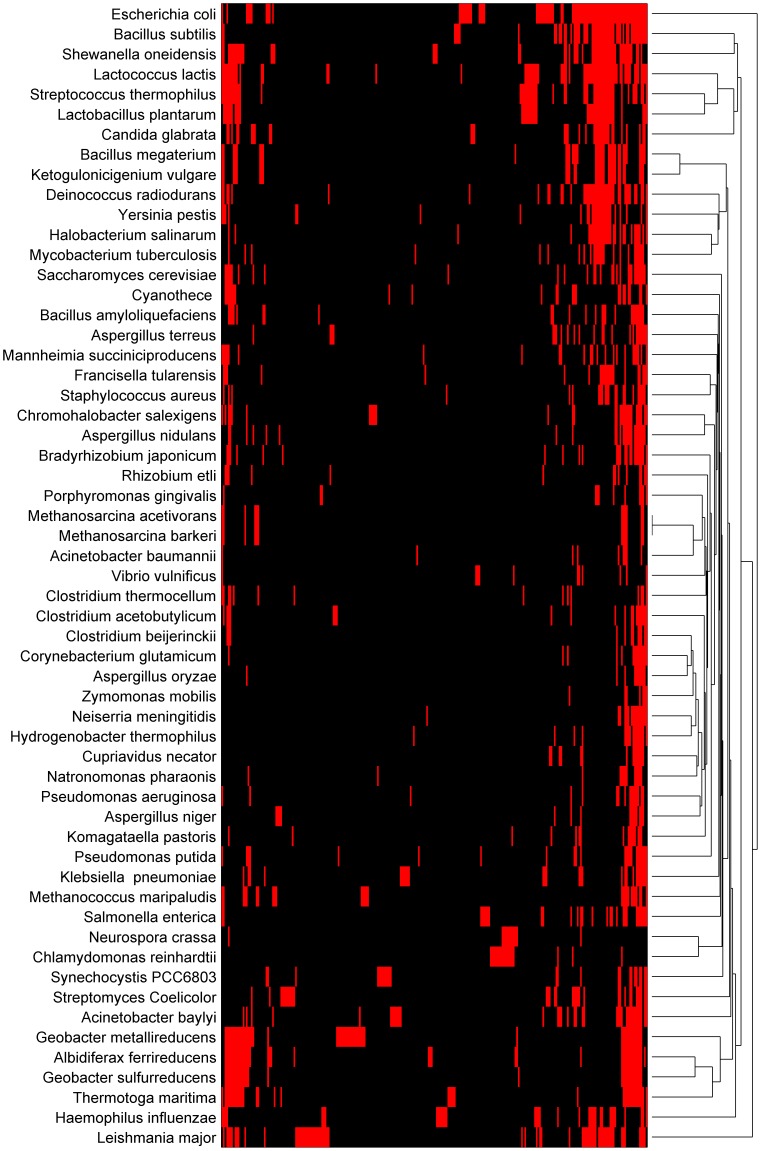
Heat map and dendrogram showing hierarchical clustering of species based on media compositions. Red bars indicate compounds that occur in at least one medium for that species. Black bars indicate compounds that do not appear in any media for that species. This figure was generated using the Statistics Toolbox in Matlab.

We used hierarchical clustering of pairwise Euclidean distance between binary vectors of compound inclusion in a medium (e.g., an entry is 1 if a given chemical is included in a medium, or 0 otherwise) to investigate the relationship between organisms in MediaDB based on published growth-supporting media. Figure 3 presents a heat map of chemical species in media, created from MediaDB data. The heat map shows bands of high-frequency compounds on the right side of the map and clusters of moderately frequent compounds on the left side; these compound groups are dominated by salts found in stock solutions and L-form amino acids, respectively. The overall sparsity of the heatmap reflects the fact that most compounds occur only once or twice across all species.

**Figure 4:**
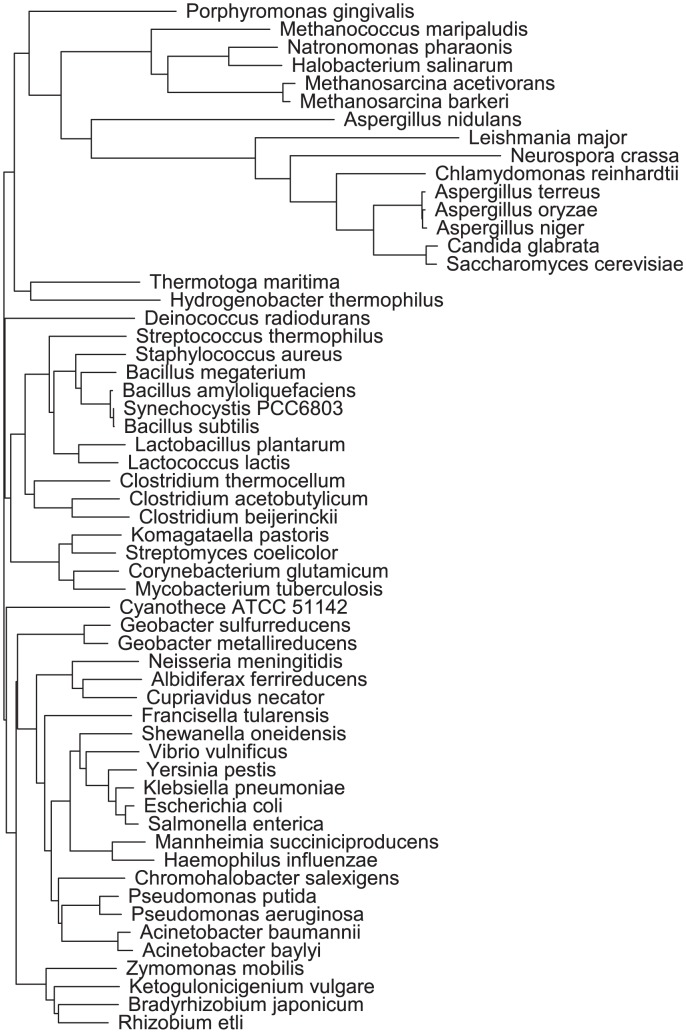
Phylogenetic tree of 16S rRNA sequences for species in MediaDB. Phylogeny was inferred from a CLUSTAL W alignment generated in the Biology Workbench using 16S rRNA sequences from the SILVA database.

We compared this compound similarity tree (Figure 3) to a 16s rRNA phylogenetic tree constructed in the Biology Workbench [46]–[49] (Figure 4) and found that there was little overlap between genetic similarity and compound similarity. Aside from the two *Methanosarcina* species, which were grown in the same exact media, we observed few parallels between these two trees. Three species in the taxonomic order *Lactobacillales*—*Lactococcus lactis, Lactobacillus plantarum*, and *Streptococcus thermophilus*—clustered closely together in both trees, but the majority of organisms that formed tight clusters in one tree did not show the same closeness in the other tree. For example, the four *Aspergilli—A. nidulans, A. niger, A. oryzae*, and *A. terreus*—were close in terms of phylogenetic distance, but dissimilar with respect to their media compounds. On the other end of the spectrum, *Corynebacterium glutamicum*, *A. oryzae, Clostridium beijerinckii*, and *Zymomonas mobilis* show high compound similarity with one another, but are far apart phylogenetically. This observation could be an indication that phylogeny does not correlate to similarity in media formulations, but a more parsimonious explanation is that the data in MediaDB reflect the literature bias towards positive growth results. Due to this lack of negative growth results (i.e. information on what an organism *does not* grow on, which is typically omitted by researchers), we are unable to assert that any organism is incapable of growth in another's media based soley on comparisons of the collected data in MediaDB. This knowledge gap suggests a need for for futher experimental study of the relationship between phylogenetic distance and nutritional requirements for growth. Thus, information available in MediaDB describes whether a given medium has been reported to support a microbe's growth, and may be useful for generating hypotheses of possible media formulations for future experimental efforts. Our analysis also revealed clusters of organisms with high media composition similarity (Figure 3) that do not have a clear connection to observed biology. With further investigation, these similarities could reveal more complex biological relationships that do not fall under the obvious prisms of genetic or environmental similarity. MediaDB will support such comparative studies as the resource continues to grow.

### Future development

#### Community-contributed growth conditions

MediaDB currently contains 57 microbial species, but the scope of the fully-sequenced microbial world is much larger and continues to grow. We intend to expand the breadth of organisms and growth conditions in MediaDB by allowing users to submit their own experimentally verified, defined growth conditions. At this time, we encourage users to submit growth conditions for our review through direct contact with the authors (mediadb@systemsbiology.org), but expect to create an input form that encourages groups to add new data directly through the website.

#### Analysis tool development

We have demonstrated the potential for media-based comparative analysis using MediaDB with E. coli and M. acetivorans; however, we have designed MediaDB to support future development of additional tools to support research efforts. We have also made the entire database schema and its contents available for download to further facilitate tool development by MediaDB users. As such tools are developed in our group and others, we will integrate these tools into the website to assist users in their analyses.

## Discussion and Conclusions

We present MediaDB, a manually curated database of defined media that have been used to cultivate organisms with sequenced genomes. Our database offers several important new capabilities for researchers through the following features: 1) brings together literature sources of experimentally verified media formulations into a centralized database; 2) contains chemically defined media, so that every compound can be linked to known metabolic pathways in metabolic network models, and so that every formulation is repeatable; 3) links with compound identifiers in existing databases for simple, repeatable and automatable cross-referencing with other sources; 4) focuses on organisms with existing *in silico* models, both encouraging researchers to use and improve such models and providing multiple media conditions to support the iterative development of *in silico* models; 5) serves as a set of organism-specific media conditions to help improve automated metabolic reconstruction methods by replacing more generic media formulations; 6) includes only species with fully-sequenced genomes to ensure that all media formulations can be tied back to genomic data; and 7) is a publically available resource that we expect will grow and increase in usage as growth conditions for more organisms are added. We anticipate that MediaDB will support the investigation of the relationship between organism growth media formulations and genomic information, and facilitate efforts to model microbial metabolism.

### Availability and requirements

The MediaDB database is a publically accessible resource, available through the Institute of Systems Biology (ISB) website at https://mediadb.systemsbiology.net. The ISB infrastructure provides a stable server platform to allow for long term maintenance of MediaDB. To submit data for upload into MediaDB, or for general questions and information, please contact the authors at mediadb@systemsbiology.org.

## Supporting Information

Figure S1 **Full MediaDB schema.** Dashed lines indicate foreign key relationships, oriented such that arrows point towards the referenced primary key. Each table is represented by a box headed by the table name and described by a list of column names and column types. This diagram was created using MySQL Workbench (www.mysql.com/products/workbench).
(PDF)

Table S1 **Full compound frequency analysis results.** The “Organism Compound Lists” worksheet lists the full set of compounds that appear in at least one media formulation for each organism species. The “Compound Frequencies” worksheet lists every compound that appears in at least one media formulation and the number of organism species known to utilize that compound (frequency). The “Organism Compound Numbers” lists every species and the number of compounds that appear in at least one media formulation for that species.
(XLSX)

File S1 **Model simulation on known media.** The compressed folder contains the *E. coli* model used for our simulations and an example Matlab script (growEcoliOnMedia.m) that demonstrates how to simulate growth of the model on media from MediaDB. This file simulates growth of *E. coli* on 11 different carbon sources corresponding to 11 different media in MediaDB.
(ZIP)
