# Supplementary figures and images for: MediaDB: A Database of Microbial Growth Conditions in Defined Media
Source: PLoS One. 2014 Aug 6;9(8):e103548. doi: 10.1371/journal.pone.0103548 (PMC4123892; doi:10.1371/journal.pone.0103548)

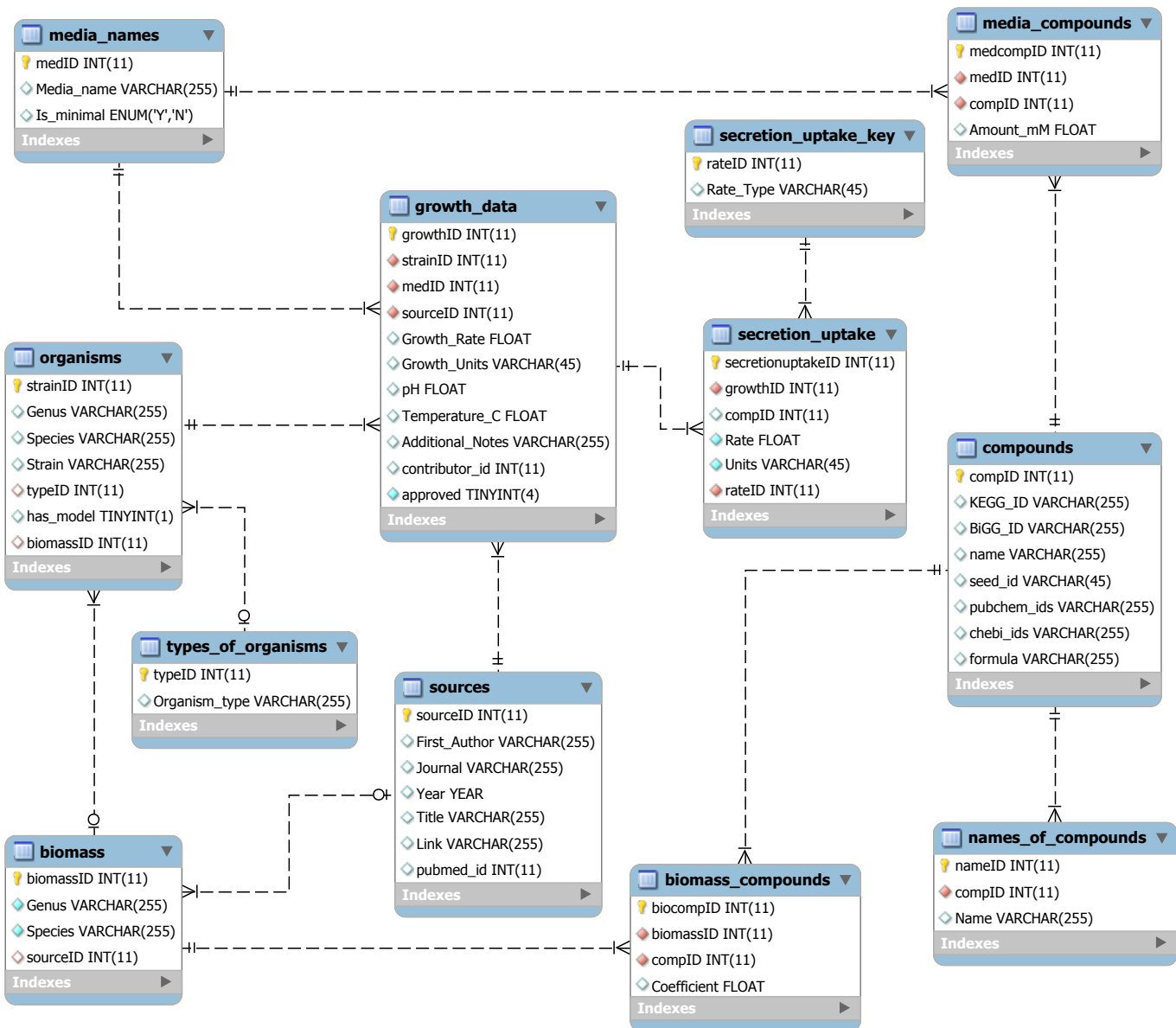

Supplement: Figure S1 — Full MediaDB schema. Dashed lines indicate foreign key relationships, oriented such that arrows point towards the referenced primary key. Each table is represented by a box headed by the table name and described by a list of column names and column types. This diagram was created using MySQL Workbench (www.mysql.com/products/workbench). (PDF) [file pone.0103548.s001.pdf]
